# No evidence for UV-based nest-site selection in sticklebacks

**DOI:** 10.1186/1742-9994-3-17

**Published:** 2006-11-14

**Authors:** Ricarda Modarressie, Theo CM Bakker

**Affiliations:** 1 Institut für Evolutionsbiologie und Ökologie, University of Bonn, An der Immenburg 1, D-53121 Bonn, Germany

## Abstract

**Background:**

Nests are built in various animal taxa including fish. In systems with exclusive male parental care, the choice of a nest site may be an important component of male fitness. The nest site may influence male attractiveness as a mate, and male, embryo, and juvenile survival probabilities. Reproductively active three-spined stickleback males establish and defend a territory in which they build a nest. Territories can differ remarkably in qualities that influence male and female reproductive success like predation risk or abiotic factors such as dissolved oxygen concentration or lighting conditions. The latter may be important because in sticklebacks the extended visual capability into the ultraviolet (UV) wave range plays a role in female mate choice. Males are thus expected to be choosy about the habitat in which they will build their nest.

**Results:**

We tested nest-site choice in male three-spined sticklebacks with respect to different UV lighting conditions. Reproductively active males were given the simultaneous choice to build their nest either in an UV-rich (UV+) or an UV-lacking (UV-) environment. Males exhibited no significant nest-site preferences with respect to UV+ or UV-. However, larger males and also heavier ones completed their nests earlier.

**Conclusion:**

We found that UV radiation as well as differences in luminance had no influence on nest-site choice in three-spined sticklebacks. Males that built in the UV-rich environment were not different in any trait (body traits and UV reflection traits) from males that built in the UV-poor environment. There was a significant effect of standard length and body mass on the time elapsed until nest completion in the UV experiment. The larger and heavier a male, the faster he completed his nest. In the brightness control experiment there was a significant effect only of body mass on the duration of nest completion. Whether nest building preferences with respect to UV lighting conditions are context dependent needs to be tested for instance by nest-site choice experiment under increased predation risk.

## Background

Nests are built in various animal taxa including arthropods, birds, mammals and fish. These nests serve mainly as a place for raising the brood [1], living in, or as a shelter, but nests as well as nest sites can also function in mate attraction [2,3]

In three-spined sticklebacks, *Gasterosteus aculeatus *L., Rowe *et al*. [4] identified microspectrophotometrically a fourth UV-sensitive visual pigment maximally absorbing (λ_max_) at around 360 nm, in addition to the three already known photopigments with λ_max _of 435 nm, 530 nm and 605 nm [5]. Reflectance measurements of reproductively active male sticklebacks indicated that they possess UV-reflective regions on their body surface [6]. Furthermore, female mate choice experiments in sticklebacks demonstrated a significant influence of UV light on mate preferences [7,8]. Also in shoal-choice experiments sticklebacks showed preferences for shoals that reflect in the UV over shoals that did not [9].

Reproductively active three-spined stickleback males show a typical nuptial coloration consisting of a red throat and a blue iris [10,11]. In the reproductive phase, males construct a tunnel-shaped nest from filamentous algae through which females can pass during spawning [12]. After spawning the female leaves the eggs to the care of the male. The male oxygenates the eggs by fanning and defends them against predators.

Nest sites may differ in quality and safety. Despite preferring male body characteristics such as the redness of the throat [13,14], female sticklebacks may base their mate choice indirectly on the quality of the territory or on nest characteristics *per se *[15,3,18]. Because reproductive success of both sexes depends strongly on embryo survival in the nest which relates to habitat properties, one would expect males to be choosy about the nest site. Such a choosiness was found in sticklebacks with regard to predator-induced nest-site choice [19], dense vegetation [20], and/or nest concealment [21,20]. But to our knowledge the influence of different environmental lighting condition on nest-site choice have not been considered thus far.

Male attractiveness to females is enhanced through their UV reflection [7,8]. One therefore may expect males to build their nests in an UV-rich environment where these reflections are most conspicuous. On the other hand, males should minimize predation risk, and one would thus expect males to build nests in an UV-poor environment, where males are less conspicuous.

We tested nest-site choice of male sticklebacks when offered an UV-rich and an UV-poor environment in the absence of predation risk.

## Results

### UV and nest-site choice

Males exhibited no significant nest-site preference for either the UV-rich (UV+) or the UV-lacking (UV-) environment: 15 males built their nests in the UV+ compartment and 14 in the UV- one (chi-square test, χ^2^_1 _= 0.034, p = 0.853). Males that built their nest in UV+ and those that built in UV- did not differ significantly with respect to standard length (UV+: mean ± s.d. = 4.45 cm ± 0.25; range 4.1 – 4.9; UV-: 4.30 cm ± 0.21; range 4.0 – 4.7), body mass (UV+: mean ± s.d = 1.19 g ± 0.22; range 0.88 – 1.53; UV-: 1.07 g ± 0.19; range 0.77 – 1.33), nor body condition (UV+: mean ± s.d = 1.34 ± 0.11; range 1.19 – 1.57; UV-: 1.33 ± 0.13; range 1.11 – 1.52) (Mann-Whitney U test, N_1 _= 15, N_2 _= 14, U = 66.5, p = 0.088; t test, t = 1.696, df = 27, p = 0.101, t test, t = 0.288, df = 27, p = 0.776, respectively).

**Figure 1:**
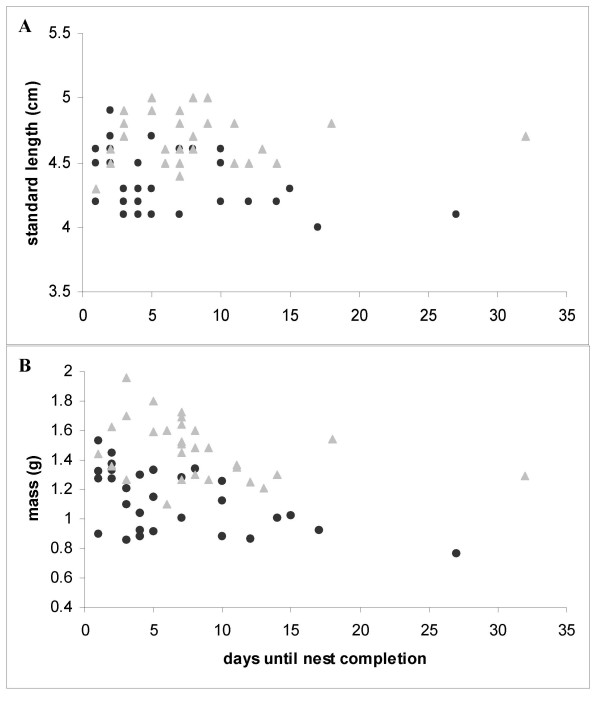
Relationship between time until nest completion (days after introduction) and (a) standard length (cm) and (b) body mass (g) in the UV-choice experiment (black dots) and the brightness control experiment (grey triangles).

Time until nest completion did not differ significantly between males that built their nest in the UV-rich environment and males that built in the UV-lacking environment (UV+: mean ± s.d = 5.8 days ± 4.02, UV-: 7.2 days ± 7.44; Mann-Whitney U test, N_1 _= 15, N_2 _= 14, U = 103, p = 0.93). But days until nest completion were negatively correlated with males' standard length (Fig. 1a) and body mass (Fig. 1b). The larger and heavier the male was, the earlier the nest was completed (Spearman rank correlation coefficient, r_s _= -0.388, N = 29, p = 0.037 and r_s _= -0.467, N = 29, p = 0.011, respectively). No significant correlation was found with respect to body condition (r_s _= -0.238, N = 29, p = 0.214).

Males that built their nest in UV+ did not visit the UV+ side significantly more often during the choice phase compared with males that built in UV- (UV+: mean ± s.d = 0.56 ± 0.23, UV-: 0.40 ± 0.29; Mann-Whitney U test, N_1 _= 15, N_2 _= 14, U = 64, p = 0.077). The side which was visited first after introduction did also not predict the side where males would build their nest (11 out of 29 males built on the side which was visited first: chi-square test, χ^2^_1 _= 1.69, p = 0.194).

### Reflectance measurements

Males that built in UV+ did not differ significantly in their UV-chroma from males that built in UV-, neither with respect to region 1 (operculum) nor region 2 (above anus) (t test; t = -1.098, df = 19, p = 0.286 and t test; t = -0.771, df = 19, p = 0.45, respectively). UV-chroma in region 2 was negatively correlated with physical condition (Pearson correlation coefficient: r_p _= -0.416, N = 23, p = 0.048). In region 1, UV-chroma and physical condition only tended to correlate (r_p _= -0.369, N = 23, p = 0.083). In both regions no significant correlations were found between UV-chroma and standard length or body mass (all p > 0.13).

Also no significant correlations were found between body characteristics and wavelength of UV-peak reflectance (hue) in both regions (all p > 0.14). Males that built in UV+ did not differ significantly from males that built in UV- with respect to hue measured in both regions (region 1: t test; t = -0.75, df = 19, p = 0.462; region 2: Mann-Whitney U test, N_1 _= 12, N_2 _= 9, U = 42.5, p = 0.413).

Furthermore, UV-contrast in regions 1 and 2 did not differ significantly between males that built in the UV+ or UV- environment (Mann-Whitney U test, N_1 _= 9, N_2 _= 12, U = 46, p = 0.57; t test, t = -0.477, df = 19, p = 0.639, respectively). No significant correlations between UV-contrast and male body characteristics were found, neither in region 1 nor region 2 (all p > 0.24). Only in region 2 body condition factor and UV-contrast tended to correlate negatively (Spearman rank correlation coefficient, r_s _= -0.394, p = 0.078).

The nests did not reflect any UV as qualitatively judged from photographs taken from the nests.

### Brightness control

In the control experiment, males also exhibited no significant nest-site preference for either the brighter (ND1) or the darker (ND2) environment: 17 males built their nest in the ND1 compartment and 12 in the ND2 one (chi-square test, χ^2^_1 _= 0.862, p = 0.353). The males that built their nest in the ND1-side and those which built in the ND2-side did not differ significantly with respect to standard length (ND1: mean ± s.d. = 4.69 cm ± 0.20; range 4.3 – 5.0; ND2: 4.65 cm ± 0.18; range 4.4 – 5.0), body mass (ND1: mean ± s.d = 1.50 g ± 0.22; range 1.10 – 1.80; ND2: 1.41 g ± 0.20; range 1.21 – 1.96), nor body condition (ND1: mean ± s.d = 1.45 ± 0.12; range 1.24 – 1.64; ND2: 1.40 ± 0.13; range 1.24 – 1.59) (Mann-Whitney U test, N_1 _= 17, N_2 _= 12, U = 89.0, p = 0.586; t test, t = 1.119, df = 27, p = 0.273, t test, t = 1.147, df = 27, p = 0.261, respectively).

Time until nest completion did not differ significantly between males that built their nest in the ND1 environment and males that built in the ND2 environment (ND1: mean ± s.d = 8.47 days ± 7.29; ND2: 7.83 days ± 3.66; t test, t = 0.278, df = 27, p = 0.783). But days until nest completion was negatively correlated with male body mass (Fig. 1b; Spearman rank correlation coefficient, r_s _= -0.437, N = 29, p = 0.018). No significant correlation was found with respect to male standard length (Fig. 1a) nor body condition (r_s _= -0.034, N = 29, p = 0.86 and r_s _= -0.235, N = 29, p = 0.22, respectively).

The side which was visited first after introduction did also not predict the side where males would build their nest (12 out of 29 males built on the side which was visited first: chi-square test, χ^2^_1 _= 0.862, p = 0.353).

## Discussion

The present study is the first to examine nest-site preferences in fish and in particular in three-spined sticklebacks with respect to different environmental UV-conditions. Contrary to expectation, sticklebacks showed no preference at all either for an UV-rich or an UV-lacking environment to build their nest in. UV radiation therefore seems to have no influence on nest-site choice in this population despite the fact that UV reflections play a role in social interactions such as female mate choice [7,8], male mate choice (I. P. Rick and T. C. M. Bakker unpublished data), and in shoaling behaviour [9] in this and other stickleback populations. The sample sizes in these studies were comparable or even lower than those of the present study.

Because the UV filters not only differed in the wave range transmitted but also in luminance, the UV-rich side was also the brighter environment. The lack of a nest-site preference may therefore have been due to a trade-off between UV radiation and brightness in nest-site choice. Such a trade-off was found for shoal choice in the same stickleback population [9]. When such a trade-off exists, one would expect that males which chose the brighter UV+ side differed from males which preferred the darker UV- side for nest building. However, no significant difference in whatsoever male characteristic measured could be assessed including body characteristics such as standard length, body mass, condition factor, and UV body-colour characteristics of two body regions such as UV-hue, -chroma, and -contrast.

Furthermore, the results of the brightness control experiment, where UV was present on both sides, revealed no preference for either the brighter or the darker nest-site environment. A trade-off between UV radiation and luminance in nest-site choice seems therefore unlikely. Possibly in another context like enhanced predation risk or the presence of ripe females, males may show a preference for a particular nest site.

Objections that males built their nest on that site which they visited first could be rejected. Moreover, the side in which the nest was built had not been visited significantly more frequently.

Larger and/or heavier males used significantly less time until nest completion, at least in the UV choice experiment. This also applied for heavier but not for larger males in the brightness control experiment. These findings agree with data from a field study of a Swiss freshwater population in which there was also a negative correlation between body size and mass of courting males with date in the breeding season [22]. We cannot discriminate whether larger males were more ready to start their breeding cycle than smaller ones and therefore started nest building earlier, or whether larger males were better in nest building once started. The time needed for nest building did not significantly differ between the UV-rich and the UV-poor environments.

Surprisingly, male body condition was negatively correlated with UV-chroma in region 2 (on the operculum), and tended to do so in region 1 (above anus). Thus males in poorer body condition showed the highest UV-chroma values. We have no interpretation for this, but all correlations will be non-significant when Bonferroni corrections are applied. In contrast to an earlier study [6], we found no significant correlation between the condition factor and UV-contrast. UV-hue was not significantly correlated to any of the body characteristics.

## Methods

### Experimental subjects

Several hundred three-spined sticklebacks, *Gasterosteus aculeatus *L., were caught with minnow traps before the start of the breeding season in 2005 from a shallow pond near Euskirchen, Germany (50°38'N/6°47'E). The pond is located in a small woodland. Because of only sparse vegetation at the shore line, it is exposed to full sunlight penetration throughout the year. The fish were released into two outdoor stocking tanks (volume 700l; provided with tap water, flow rate of 3 litres per minute and air ventilation). To guarantee full penetration of UV-rich sunlight, stocking tanks were cleaned regularly. Fish were fed daily *ad libitum *on a diet of frozen chironomid larvae.

Males showing developing nuptial coloration were singled out with a hand net from the stocking tanks and released individually into bare small aquaria (30 × 20 × 20 cm, l × w × h) in the laboratory. Illumination was provided by fluorescent tubes (True Light, Natural Daylight 5500, 36 Watt, 1200 mm) hanging 15 cm above the water surface. These lights contain a proportion of UV similar to natural skylight. The fish were kept under a 16:8 h light-dark regime at 17 ± 2°C. As before, fish were fed daily *ad libitum *with frozen chironomid larvae. Each male was sexually stimulated with a ripe female enclosed in a transparent plastic box presented in front of the males' aquaria for ten minutes daily.

### Experimental set-up

**Figure 2:**
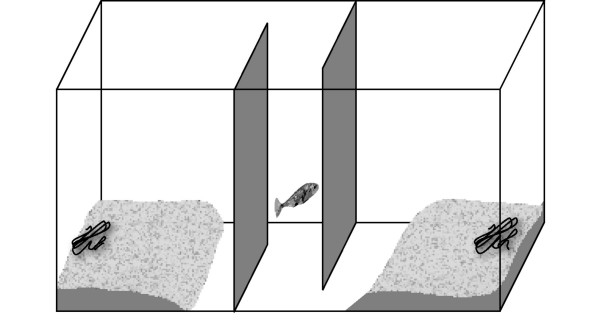
Experimental set-up. The choice aquarium was divided into two equal compartments which were separated by a sluice formed by two opaque plastic partitions. One side was covered by an UV-transmitting (or ND1), the other side by an UV-blocking filter (or ND2).

After one week of sexual stimulation, males were given simultaneously the choice to build their nests either in an UV-rich or UV-lacking environment. The choice aquarium (Fig. 2; 100 × 45 × 45 cm, l × w × h) was divided into two equal-sized compartments separated by a kind of sluice in the middle of the tank. The sluice consisted of two opaque, grey plastic partitions (each 30 × 30 cm, l × h) separated by 8 cm, one leaving an opening at the front wall, the other at the back wall. In this way test fish could freely visit both sides but had no direct view of both compartments. The aquarium was filled with tap water to a level of 15 cm, and the bottom was covered with fine gravel. In both compartments equal amounts of filamentous algae (1.56 g ± 0.04 g) was provided as nest material. Again, illumination was given by a fluorescent tube (True Light, Natural Daylight 5500, 36 Watt, 1200 mm) hanging 25 cm above the water surface. The top of one compartment was covered by an UV-transmitting filter (GS-2485, Röhm Plexiglas, Germany) whereas the other one was covered by an UV-blocking filter (GS-233, Röhm Plexiglas, Germany). To prevent potentially confounding, external influences, the walls of the aquarium were fitted out with grey, opaque plastic partitions which reflected moderately in the UV-A range.

Test fish were gently taken out of their holding tanks with a hand net and introduced into the sluice of the test aquarium. The side which was visited first was noticed. Three times daily, between 9:00–17:00 hours, it was scored whether the male was at the UV-rich or UV-lacking side or in the sluice. After the last positison check of the day, test fish were fed with frozen chironomid larvae in the sluice.

After completion of the nest, indicated by the male creeping through it, test fish were weighed to the nearest milligram and standard length was measured. A total of 33 males were tested of which 4 were disregarded because they failed to complete their nest or even to commence nest building within 25 days after introduction in the choice aquarium. Male condition factor (CF) was calculated after CF = 100 × *W*/*SL*^3 ^(where *W *is body mass (g), and *SL *is standard length (cm); [23]). Trials were conducted between 20 May and 4 August 2005.

The UV reflectance of 23 of the test males was measured, directly after the choice test, at two body regions (at the operculum, and above the anus; for details see [6]) using an Avantes USB-2000 fibre-optic spectrometer. A bifurcated 200 micron fibre-optic probe, with unidirectional illumination and recording, was held at a 90° angle to the body surface. Illumination was given by a deuterium-halogen-light source (Avantes DH-2000, 215–1700 nm). A darkened pipette tip was mounted on the probe end in order to exclude ambient light and to measure reflectance at a fixed distance of 4 mm from the body surface [24]. Reflectance intensity over the range of 310–710 nm was recorded relative to a 99% Spectralon white-standard. Data were recorded with Spectrawin 5.1 (Avantes, Netherlands) and imported into Microsoft Excel. The wavelength of peak reflectance in the UV (hue), UV-chroma (% UV reflectance of total reflectance), and the UV-contrast C (difference in intensity between the UV peak and the lowest value of the reflection curve; see [6]) were calculated from the spectral data [25]. The average value of ten measurements of both body regions per fish were used for statistical analysis.

The nests of 5 males were taken out of the choice tank, put into a dish filled with water, and photographed under UV light conditions using a Sony DSC-F707 digital camera through a combination of two filters (BG 38 and UG 1, Schott glass, Darmstadt, Germany) (see [26]).

### Brightness control

Due to a 18% difference in spectral transmission between the UV(+) and UV(-) filters, we conducted a control experiment to test whether nest-site choice is influenced by a difference in brightness. The experimental procedure was exactly the same as mentioned above, except that the UV(+) and UV(-) filters were replaced by two neutral density (ND1 and ND2, Cotech 298 and Lee 209, Zilz, Germany) filters. ND1 and ND2 filters differed approximately 34% in their quantitative transmission (see [8]), which is nearly twice as much as between the UV treatment filters. The neutral density (ND) filters were transmittive for wavelengths between 300 nm and 800 nm, but altered luminance independent of hue. We tested a total of 30 males of which one was discarded because it failed to commence nest-building within 25 days. Trials were run between 24 may and 07 august 2006 using males that had been caught before the start of the 2006 breeding season from a shallow pond near Euskirchen, Germany.

### Statistical analysis

All analyses were performed using SPSS 11.0 for Windows. When data were not normally distributed according to the Kolmogorov-Smirnov test with Lilliefors correction, non-parametric statistics was applied. Given p-values are two-tailed throughout.

### Ethical note

Animal care and experimental procedures were in accordance with the legal requirements of Germany. No additional license was required for this study.

## Competing interests

The author(s) declare that they have no competing interests.

## Authors' contributions

RM designed and carried out the experiments, performed the statistical data analysis, and drafted the manuscript. TCMB participated in its design and coordination, and helped to draft the manuscript. All authors read and approved the final manuscript.
